# Prevalence of and factors associated with anaemia in women of reproductive age in Bangladesh, Maldives and Nepal: Evidence from nationally-representative survey data

**DOI:** 10.1371/journal.pone.0245335

**Published:** 2021-01-07

**Authors:** Md. Ashfikur Rahman, Md. Sazedur Rahman, Muhammad Aziz Rahman, Ewa A. Szymlek-Gay, Riaz Uddin, Sheikh Mohammed Shariful Islam

**Affiliations:** 1 Development Studies Discipline, Khulna University, Khulna, Bangladesh; 2 Statistics Discipline, Khulna University, Khulna, Bangladesh; 3 School of Nursing and Healthcare Professions, Federation University Australia, Berwick, Victoria, Australia; 4 School of Nursing and Midwifery, La Trobe University, Melbourne, Victoria, Australia; 5 Institute for Physical Activity and Nutrition, School of Exercise and Nutrition Sciences, Deakin University, Geelong, Victoria, Australia; ESIC Medical College & PGIMSR, INDIA

## Abstract

**Background:**

Anaemia is a significant public health problem in most South-Asian countries, causing increased maternal and child mortality and morbidity. This study aimed to estimate the prevalence of and factors associated with anaemia in women of reproductive age in Bangladesh, Maldives, and Nepal.

**Methods:**

We used the nationally-representative Demographic and Health Surveys Program data collected from women of reproductive age (15–49 years) in 2011 in Bangladesh (n = 5678), 2016 in Maldives (n = 6837), and 2016 in Nepal (n = 6419). Anaemia was categorized as mild (haemoglobin [Hb] of 10.0–10.9 g/dL for pregnant women and 11.0–11.9 g/dL for non-pregnant women), moderate (Hb of 7.0–9.9 g/dL for pregnant women and 8.0–10.9 g/dL for non-pregnant women), and severe (Hb <7.0 g/dL for pregnant women and *<*8.0 g/dL for non-pregnant women). Multinomial logistic regression analyses were used to identify factors associated with anaemia.

**Results:**

The prevalence of anaemia was 41.8% in Bangladesh, 58.5% in Maldives, and 40.6% in Nepal. In Bangladesh, postpartum amenorrhoeic, non-educated, and pregnant women were more likely to have moderate/severe anaemia compared to women who were menopausal, had secondary education, and were not pregnant, respectively. In Maldives, residence in urban areas, underweight, having undergone female sterilization, current pregnancy, and menstruation in the last six weeks were associated with increased odds of moderate/severe anaemia. In Nepal, factors associated with increased odds of moderate/severe anaemia were having undergone female sterilization and current pregnancy.

**Conclusion:**

Anaemia remains a significant public health issue among 15-49-year-old women in Bangladesh, Maldives, and Nepal, which requires urgent attention. Effective policies and programmes for the control and prevention of anaemia should take into account the unique factors associated with anaemia identified in each country. In all three countries, strategies for the prevention and control of anaemia should particularly focus on women who are pregnant, underweight, or have undergone sterilization.

## Introduction

Anaemia is a significant public health problem worldwide, especially in South Asia [1–5]. Globally, half a billion women of reproductive age are affected by anaemia, with the largest proportion living in South Asia [6]. Although the worldwide prevalence of anaemia decreased from 33% to 29% between 1995 and 2011 among non-pregnant women, and from 43% to 38% among pregnant women, it remains a major public health threat requiring urgent attention to meet one of the six recent Global Nutrition Targets of reducing anaemia by 50% in women of reproductive age by 2025 [6]. Despite substantial economic growth, South Asia has made little progress in reducing anaemia. A WHO study has suggested that over 50% of women of reproductive age are affected by anaemia in some South-Asian countries [7]. In 2012, South Asia also accounted for 38% of the world’s years lost to disability associated with anaemia [8, 9].

Anaemia is an indicator of poor nutrition and health [6] and is associated with decreased work productivity due to reduced work capacity, which in turn leads to reduced personal income and substantial economic losses to the country [4, 10]. Anaemia contributes to increased risk of miscarriages and premature birth in mothers, and low birth weight, suboptimal growth and impaired learning in children [11], which may also persist into adulthood. The annual physical productivity losses caused by anaemia are approximately $3.64 per capita, which is 0.81% of Gross Domestic Product in ten selected developing countries [12]. This small individual loss may aggregate the effect, particularly in developing countries in which physical labour is dominant [10] and accrues billions of dollars in human capital. In South Asia, this productivity loss is estimated to cost $4.2 billion annually [12].

The WHO estimates that approximately half of all anaemia cases is due to iron deficiency with other contributing factors including genetic disorders, chronic infections, and micronutrient deficiencies [2, 3, 8, 13]. Other factors such as socioeconomic, demographic, anthropometric, and lifestyle factors have also been shown to play a role in the development of anaemia [3, 4, 14, 15]. Several studies have been conducted on anaemia among women in South Asia, showing an association with multiple risk factors [3, 4, 15, 16]. However, these studies did not categorize the severity of anaemia and its association with risk factors. Therefore, in this study, we estimated the prevalence of anaemia and identified factors associated with anaemia in women of reproductive age (15–49 years) in Bangladesh, Maldives, and Nepal.

## Methods

### Data sources

We used data from the 2011 Bangladesh Demographic and Health Survey conducted from 8 July 2011 to 27 December 2011, the 2016 Nepal Demographic and Health Survey carried out from 19 June 2016 to 31 January 2017, and the 2016 Maldives Demographic and Health Survey conducted from 17 March 2016 to 27 November 2017 –these data were collected as part of the Demographic and Health Surveys Program and are publicly available at https://dhsprogram.com/. Each dataset included values for haemoglobin (Hb) concentrations or data for the presence of anaemia along with socioeconomic, demographic, anthropometric, and lifestyle data for women of reproductive age (15–49 years). The details of the survey process and sampling, household selection, blood collection, and Hb determination have been reported for each country [17–19]. In brief, all three surveys used a multi-stage cluster sampling approach. Capillary blood samples were collected using a finger-prick method, and Hb concentration was determined using a battery-operated photometer and a disposable microcuvette (HemoCue rapid testing methodology) [17–19].

### Dependent variable–anaemia

Women of reproductive age (15–49 years) were included in this analysis if they had data for Hb concentration or data for the presence of anaemia. We used the adjusted Hb concentration available in the datasets. Hb concentrations were adjusted for all three countries for altitude and smoking as described previously [17–20]. Anaemia was defined according to the WHO classification as Hb <11 g/dL for pregnant women and <12 g/dL for non-pregnant women [21]. Anaemia was further categorized as mild (Hb of 10.0–10.9 g/dL for pregnant women and 11.0–11.9 g/dL for non-pregnant women), moderate (Hb of 7.0–9.9 g/dL for pregnant women and 8.0–10.9 g/dL for non-pregnant women, and severe (Hb <7.0 g/dL for pregnant women and *<*8.0 g/dL for non-pregnant women) [21]. However, due to a small number of women presenting with severe anaemia, the moderate and severe anaemia categories were combined into a moderate/severe category (Hb <10.0 g/dL for pregnant women and <11.0 g/dL for non-pregnant women) [21].

### Independent variables–factors associated with anaemia

Factors were chosen based on previous literature reporting the risk of developing anaemia in women in low- and middle-income countries including Nepal, Bangladesh, Pakistan, India, and Tanzania [3, 4, 10, 14, 15, 22–24]. Socioeconomic and demographic variables included: participants’ current age; place of residence; education level; occupation; husband’s education level; husband’s occupation; sources of drinking water; toilet facilities; and household wealth status. Reproductive characteristics included: total number of children ever birthed; age at first birth; current contraception use; birth in the last 3 years; current pregnancy status; last birth by caesarean section; currently breastfeeding; fertility status; ever had a terminated pregnancy; and menstruated in the last 6 weeks. Behavioural characteristics included cigarette smoking. Weight and height were measured using the Demographic and Health Surveys Program protocols. Body mass index (BMI) was calculated as weight in kilograms divided by height in meters squared. BMI was used to identify underweight, normal weight, and overweight/obesity according to the WHO classification [25]. Self-reported age was categorized into three groups, and household wealth status was categorized into five groups based on principal component analysis [17–19], which were then recoded into three groups as poor (poorer + poorest), medium, and rich (richer + richest).

### Statistical analyses

Descriptive statistics were used to summarize background characteristics and the prevalence of anaemia for each country. Chi-square test was used to determine the associations between the independent and outcome variables where p-value of <0.05 was taken as statistically significant in each country. Predictor variables associated at p<0.05 were considered in multiple multinomial logistic regression models to capture the effect of each independent variable with the outcome variable which had three categories (no anaemia, mild, moderate/severe). Both unadjusted/crude odds ratios (cORs) and adjusted odds ratios (aORs) are reported for these analyses along with 95% confidence intervals (CIs). Effects of collinearity were checked for in all models and found to be low with variance inflation factors ≤2 for all independent variables. All statistical analyses were performed using SPSS 26 (IBM Windows Version) software. All tests were two-sided.

### Ethical statement

The study used secondary data from the Demographic and Health Surveys Program that are publicly available. The details of ethical procedures followed by the Demographic and Health Surveys Program can be found elsewhere [26].

## Results

A total of 5678 women of reproductive age from Bangladesh were included in this study. Most women were aged between 35–49 years (36.7%) and 65.3% resided in rural areas. The overall prevalence of anaemia in Bangladesh was 41.8%, with 6.8% of all women presenting with moderate/severe anaemia and 35.0% with mild anaemia. Anaemia was more prevalent in women residing in rural areas (44.2%); if the woman (46.2%) or her husband (45.5%) had no education; in women whose husband had an agricultural occupation (47.3%); in women who lived in a household with a tube well as a source of drinking water (43.2%); in women from households with no toilet (53%); and in women who were poor (48.2%), underweight (51.1%), gave birth to ≥3 children (45.1%), underwent sterilization (47.5%), were currently pregnant (49%), were currently breastfeeding (46.3%), had postpartum amenorrhea (56.4%), or did not menstruate in the last six weeks (45.1%) (p<0.001; Table 1).

**Table 1:** Characteristics of 15-49-year-old women participating in the 2011 Bangladesh Demographic and Health Survey.

General Characteristics	Total N (%)	Prevalence of Anaemia, N (%)			P*-*Value
		Moderate/severe	Mild	No Anaemia	
**Current Age**	5678				0.704
15–24 Years	1701 (30.0)	117 (6.9)	583 (34.3)	1001 (58.8)	
25–34 Years	1894 (33.4)	262 (6.8)	1355 (35.1)	2243 (58.1)	
35–49 Years	2083 (36.7)	9 (7.7)	47 (40.2)	61 (52.1)	
**Place of Residence**	5678				<0.001
Urban	1970 (34.7)	118 (6.0)	615 (31.2)	1237 (62.8)	
Rural	3708 (65.3)	270 (7.3)	1370 (36.9)	2068 (55.8)	
**Religion**	5678				0.001
Islam	5022 (88.4)	333 (6.6)	1717 (34.2)	2972 (59.2	
Hindu	623 (11.0)	54 (8.7)	255 (40.9)	314 (50.4)	
Other	33 (0.6)	1 (0.3)	13 (39.4)	19 (57.6)	
**Education Level**	5678				<0.001
No Education	1457 (25.7)	130 (8.9)	544 (37.3)	783 (53.7)	
Primary	1746 (30.8)	131 (7.5)	640 (36.7)	975 (55.8)	
Secondary	2050 (36.1)	103 (5.0)	685 (33.4)	1262 (61.6)	
Higher	425 (7.5)	24 (5.6)	116 (27.3)	285 (67.1)	
**Occupation**	5678				0.035
Agricultural	170 (3.0)	21 (12.4)	58 (34.1)	91 (53.5)	
Professional/Services	330 (5.8)	23 (7.0)	126 (38.2)	181 (54.8)	
Other/Don’t Work	5178 (91.2)	344 (6.6)	1801 (34.8)	3033 (58.6)	
**Husband’s Education Level**	5678				<0.001
No Education	1640 (28.9)	127 (7.7)	620 (37.8)	893 (54.5)	
Primary	1625 (28.6)	129 (7.9)	579 (35.6)	917 (56.4)	
Secondary	1612 (28.4)	89 (5.5)	538 (33.4)	985 (61.1)	
Higher	801 (14.1)	43 (5.4)	248 (31.0)	510 (63.7)	
**Husband’s Occupation**	5678				<0.001
Agricultural	1679 (29.6)	144 (8.6)	650 (38.7)	885 (52.7)	
Non-Agricultural	2178 (38.4)	130 (6.0)	725 (33.3)	1323 (60.7)	
Business	1246 (21.9)	78 (6.3)	413 (33.1)	755 (60.6)	
Professional/Services	364 (6.4)	25 (6.9)	114 (31.3)	225 (61.8)	
Other/Don’t Work	211 (3.7)	11 (5.2)	83 (39.4.2)	117 (55.5)	
**Sources of Drinking Water**	5678				<0.001
Tube Well	4499 (79.2)	306 (6.8)	1636 (36.4)	2557 (56.8)	
Piped Water	619 (10.9)	37 (6.0)	157 (25.4)	425 (68.7)	
Other	560 (9.9)	45 (8.0)	192 (34.3)	323 (57.7)	
**Toilet Facilities**	5678				<0.001
Flush Toilet	9588 (16.9)	52 (5.4)	247 (25.8)	659 (68.8)	
Pit Latrine	3812 (67.1)	267 (7.0)	1381 (36.2)	2164 (56.8)	
Other	719 (12.7)	50 (7.0)	276 (38.4)	393 (54.7)	
No Toilet	189 (3.3)	19 (10.1)	81 (42.9)	89 (47.1)	
**Household Wealth Status**	5678				<0.001
Poor	2065 (36.4)	176 (8.5)	820 (39.7)	1069 (51.8)	
Medium	1078 (19.0)	73 (6.8)	391 (36.3)	614 (57.0)	
Rich	2535 (44.6)	139 (5.5)	774 (30.5)	1622 (64.0)	
**BMI**	5678				<0.001
Underweight (<18.5 kg/m^2^)	1360 (24.0)	115 (8.4)	585 (42.7)	670 (48.9)	
Normal (18.5–24.99 kg/m^2^)	3296 (58.0)	233 (6.9)	1151 (34.0)	1999 (59.1)	
Overweight/Obese (≥25.0 kg/m^2^)	1022(18.0)	29 (4.4)	181 (27.7)	444 (67.9)	
**Total Number of Children Ever Birthed**	5678				<0.001
≥3 Children	2518 (44.3)	196 (7.8)	940 (37.3)	1382 (54.9)	
1–2 Children	1588 (45.6)	148 (5.7)	857 (33.1)	1583 (61.2)	
No Children	572 (10.1)	44 (7.7)	188 (32.9)	340 (59.4)	
**Age at First Birth**	5102				0.667
15–24 Years	4863 (95.2)	328 (6.7)	1706 (35.1)	2829 (58.2)	
25–34 Years	243 (4.3)	14 (5.9)	90 (37.7)	135 (56.5)	
**Current Contraception Use**	5678				<0.001
Female Sterilization	261 (4.6)	20 (7.7)	104 (39.8)	137 (52.5)	
Using Other Method	3020 (53.2)	140 (4.6)	1036 (34.3)	1844 (61.1)	
Not Using any Method	2397 (42.2)	228 (9.5)	845 (35.3)	1324 (55.2)	
**Birth in the Last 3 Years**	5678				0.007
Yes	1431 (25.2)	111 (7.8)	537 (37.5)	783 (54.7)	
No	4247 (74.8)	277 (6.5)	1448 (34.1)	2522 (59.4)	
**Current Pregnancy Status**	5678				<0.001
Yes	357 (6.3)	81 (22.7)	94 (26.3)	182 (51.0)	
No	5321 (93.7)	307 (5.8)	1891 (35.5)	3123 (58.7)	
**Last Birth by Caesarean Section**	2267				0.031
Yes	332 (14.6)	13 (3.9)	114 (34.3)	205 (61.7)	
No	1935 (85.4)	144 (7.4)	704 (36.4)	1087 (56.2)	
**Currently Breastfeeding**	5678				<0.001
Yes	1417 (25.0)	103 (7.3)	552 (39.0)	762 (53.8)	
No	4261 (75.0)	285 (6.7)	1433 (33.6)	2543 (59.7)	
**Fertility Status**	5325				<0.001
Fecund	3814 (71.6)	185 (4.9)	1345 (35.3)	2284 (59.9)	
Pregnant	356 (6.7)	80 (22.5)	94 (26.4)	182 (51.1)	
Postpartum Amenorrhoeic	289 (5.4)	40 (13.8)	123 (42.6)	126 (43.6)	
Infecund, Menopausal	866 (16.3)	55 (6.4)	297 (34.3)	514 (59.4)	
**Ever had a Terminated Pregnancy**	5678				0.810
Yes	1286 (22.6)	93 (7.2)	449 (34.9)	744 (22.5)	
No	4392 (77.4)	295 (6.7)	1536 (35.0)	2561 (58.3)	
**Menstruated in the Last 6 Weeks**	5678				<0.001
Yes	3919 (69.0)	207 (5.3)	1373 (35.0)	2339 (59.7)	
No	1759 (31.0)	181 (10.3)	612 (34.8)	966 (54.9)	

P-value obtained from Chi-square test.

Women who had no education were 2.58 (95% CI: 1.46–4.55; p = 0.001) times more likely to have moderate/severe anaemia than women who had secondary education (Table 2). Women who were postpartum amenorrhoeic were approximately five times (aOR = 4.81, 95% CI: 1.63–14.17; p = 0.004) more likely to be moderately/severely anaemic than infecund, menopausal women. Pregnant women were more likely to be moderately/severely anaemic than non-pregnant women (aOR = 10.93, 95% CI: 3.53–33.70; p<0.001). Overweight/obesity was associated with decreased odds of moderate/severe anaemia compared to normal weight (aOR = 0.43, 95% CI: 0.21–0.92; p = 0.028). Factors associated with increased odds of mild anaemia were: women’s professional/services occupation compared to other non-agricultural occupations or current unemployment (aOR = 1.87, 95% CI: 1.14–3.03; p = 0.011); having given birth to ≥3 children compared to having given birth to no children (aOR = 1.26, 95% CI: 1.01–1.57; p = 0.038); and currently breastfeeding compared to not breastfeeding (aOR = 1.37, 95% CI: 1.12–1.67; p = 0.002) (Table 2). The following factors were associated with decreased odds of mild anaemia: non-agricultural occupation of the husband compared to no current employment or occupations other than agricultural, business or professional/services (aOR = 0.49, 95% CI: 0.27–0.91; p = 0.023); use of contraception other than sterilization compared to not using contraception (aOR = 0.68, 95% CI: 0.52–0.88; p = 0.003); and overweight/obesity compared to normal weight (aOR = 0.70, 95% CI: 0.51–0.96; p = 0.024) (Table 2).

**Table 2:** Factors associated with anaemia among women of reproductive age in Bangladesh (n = 5678).

General Characteristics	Moderate/severe Anaemia				Mild Anaemia			
	cOR [95% CI]	P-Value	aOR [95% CI]	P-Value	cOR [95% CI]	P-Value	aOR [95% CI]	P-Value
**Place of Residence**								
Rural (Ref.)	1.00		1.00		1.00		1.00	
Urban	0.73 [0.58–0.92]	0.007	1.16 [0.73–1.85]	0.521	0.75 [0.67–0.85]	<0.001	1.11 [0.88–1.40]	0.381
**Religion**								
Other (Ref.)	1.00		1.00		1.00		1.00	
Islam	2.13 [0.28–15.95]	0.462	0.63 [0.28–15.95]	0.462	0.84 [0.42–1.71]	0.537	0.37 [0.10–1.60]	0.183
Hindu	3.27 [0.43–24.92]	0.253	1.27 [0.43–24.92]	0.456	1.19 [0.58–2.45]	0.643	0.48 [0.11–2.11]	0.329
**Education Level**								
Secondary (Ref.)	1.00		1.00		1.00		1.00	
No Education	1.97 [1.25–3.11]	0.004	2.58 [1.46–4.55]	0.001	1.71 [1.34–2.17]	<0.001	1.00 [0.59–1.72]	0.989
Primary	1.60 [1.01–2.51]	0.044	1.58 [0.97–2.56]	0.064	1.61 [1.27–2.05]	<0.001	1.17 [0.72–1.90]	0.534
Higher	0.97 [0.61–1.54]	0.895	1.19 [0.44–3.25]	0.736	1.33 [1.05–1.70]	0.016	1.13 [0.74–1.75]	0.601
**Occupation**								
Other/Don’t Work (Ref.)	1.00		1.00		1.00		1.00	
Agricultural	2.04 [1.25–3.13]	0.004	1.41 [0.53–3.77]	0.492	1.07 [0.77–1.50]	0.678	1.16 [0.65–2.06]	0.616
Professional/Services	1.12 [0.72–1.75]	0.619	1.70 [0.66–4.39]	0.274	1.17 [0.93–1.48]	0.184	1.87 [1.14–3.03]	0.011
**Husband’s Education Level**								
Secondary (Ref.)	1.00		1.00		1.00		1.00	
No Education	1.69 [1.17–2.42]	0.005	0.95 [0.53–1.71]	0.873	1.43 [1.19–1.72]	<0.001	0.84 [0.54–1.31]	0.447
Primary	1.67 [1.16–2.40]	0.006	1.47 [0.89–244]	0.138	1.30 [1.08–1.56]	0.005	0.91 [0.61–1.35]	0.625
Higher	1.07 [0.73–1.57]	0.721	1.16 [0.46–2.89]	0.756	1.12 [0.93–1.35]	0.217	0.94 [0.64–1.36]	0.935
**Husband’s Occupation**								
Other/Don’t Work (Ref.)	1.00		1.00		1.00		1.00	
Agricultural	1.73 [0.91–3.29]	0.094	1.35 [0.29–6.36]	0.707	1.03 [0.77–1.40]	0.820	0.61 [0.33–1.14]	0.112
Non-Agricultural	1.05 [0.55–1.99]	0.893	1.12 [0.24–5.24]	0.883	0.77 [0.58–1.04]	0.087	0.49 [0.27–0.91]	0.023
Business	1.10 [0.57–2.13]	0.789	1.06 [0.22–5.06]	0.947	0.77 [0.57–1.05]	0.096	0.56 [0.30–1.10]	0.067
Professional/Services	1.18 [0.56–2.49]	0.660	2.26 [0.37–14.10]	0.378	0.71 [0.50–1.02]	0.067	0.75 [0.34–1.58]	0.464
**Sources of Drinking Water**								
Other (Ref.)	1.00		1.00		1.00		1.00	
Tube Well	0.86 [0.62–1.12]	0.372	0.80 [0.42–1.53]	0.494	1.08 [0.89–1.30]	0.446	1.53 [1.04–2.26]	0.031
Piped Water	0.63 [0.40–0.99]	0.044	0.91 [0.34–2.46]	0.850	0.62 [0.48-.803]	<0.001	1.32 [0.78–2.34]	0.301
**Toilet Facilities**								
No Toilet (Ref.)	1.00		1.00		1.00		1.00	
Flush Toilet	0.37 [0.21-.654]	0.001	0.52 [0.17–1.53]	0.233	0.41 [0.30–0.58]	<0.001	0.89 [0.48–1.62]	0.677
Pit Latrine	0.58 [0.35-.964]	0.036	0.73 [0.34–0.16]	0.424	0.70 [0.52–0.96]	0.024	1.09 [0.66–1.80]	0.756
Other	0.60 [0.34–1.06]	0.078	0.59 [0.24–1.45]	0.247	0.77 [0.55–1.08]	0.133	1.28 [0.72–2.28]	0.408
**Household Wealth Status**								
Medium (Ref.)	1.00		1.00		1.00		1.00	
Poor	1.39 [1.04–1.85]	0.028	1.20 [0.73–1.98]	0.481	1.21 [1.03–1.41]	0.019	1.02 [0.79–1.34]	0.866
Rich	0.72 [0.54–0.97]	0.031	1.01 [0.56–1.80]	0.986	0.75 [0.64–0.87]	<0.001	0.77 [0.58–1.02]	0.766
**BMI**								
Normal (18.5–24.99 kg/m^2^) (Ref.)	1.00		1.00		1.00		1.00	
Underweight (<18.5 kg/m^2^)	1.45 [1.14–1.84]	0.003	0.96 [0.64–1.43]	0.834	1.47 [1.29–1.68]	<0.001	1.20 [0.98–1.48]	0.085
Overweight/Obese (≥25.0 kg/m^2^)	0.55 [0.40–0.77]	<0.001	0.43 [0.21–0.92]	0.028	0.65 [0.55–0.76]	<0.001	0.70 [0.51–0.96]	0.024
**Total Number of Children Ever Birthed**								
No Children (Ref.)	1.00		1.00		1.00		1.00	
≥3 Children	1.10 [0.77–1.55]	0.606	1.31 [0.87–1.97]	0.202	1.23 [1.01–1.50]	0.039	1.26 [1.01–1.57]	0.038
1–2 Children	0.72 [0.51–1.03]	0.074	0.72 [0.51–1.03]	0.674	0.98 [0.80–1.19]	0.833	0.76 [0.80–1.19]	0.837
**Current Contraception Use**								
Not Using any Method (Ref.)	1.00		1.00		1.00		1.00	
Female Sterilization	0.85 [0.52–1.38]	0.509	1.15 [0.35–3.80]	0.814	1.19 [0.91–1.56]	0.206	1.41 [0.79–2.50]	0.245
Using Other Method	0.44 [0.35–0.55]	<0.001	0.88 [0.50–1.57]	0.663	0.88 [0.79–0.99]	0.030	0.68 [0.52–0.88]	0.004
**Current Pregnancy Status**								
No (Ref.)	1.00		1.00		1.00		1.00	
Yes	4.53 [3.40–6.03]	<0.001	10.93 [3.53–33.70]	<0.001	0.85 [0.66–1.10]	0.222	1.08 [0.59–1.95]	0.819
**Last Birth by Caesarean Section**								
No (Ref.)	1.00		1.00		1.00		1.00	
Yes	0.479 [0.27–0.86]	0.014	0.76 [0.39–1.52]	0.443	0.86 [0.67–1.10]	0.228	0.99 [0.73–1.32]	0.919
**Currently Breastfeeding**								
No (Ref.)	1.00		1.00		1.00		1.00	
Yes	1.21 [0.95–1.53]	0.125	1.31 [0.87–1.98]	0.196	1.29 [1.13–1.46]	<0.001	1.37 [1.12–1.67]	0.002
**Fertility Status**								
Infecund, Menopausal (Ref.)	1.00		1.00		1.00		1.00	
Fecund	0.76 [0.55–1.04]	0.084	1.07 [0.31–3.63]	0.916	1.02 [0.87–1.19]	0.814	1.22 [0.73–2.05]	0.450
Pregnant	4.11 [2.80–6.02]	<0.001	10.93 [3.53–33.70]	<0.001	0.89 [0.67–1.19]	0.443	1.08 [0.59–1.95]	0.819
Postpartum Amenorrhoeic	2.97 [1.89–4.66]	<0.001	4.81 [1.63–14.17]	0.004	1.69 [1.27–2.25]	<0.001	1.29 [0.78–2.15]	0.327
**Menstruated in the Last 6 Weeks**								
No (Ref.)	1.00		1.00		1.00		1.00	
Yes	0.47 [0.38–0.58]	<0.001	1.36 [0.60–3.12]	0.463	0.928 [0.82–1.10]	0.217	0.97 [0.69–1.38]	0.870

Ref: Reference; CI: Confidence Interval.

P-value obtained from multiple multinomial logistic regression models.

Table 3 shows that among 6837 women of reproductive age in Maldives, 37.1% were aged 25–34 years, 3.7% were pregnant, and 88.2% resided in rural areas. The overall prevalence of anaemia in Maldives was 58.5%, mild anaemia was present in 46.4% of all women while moderate/severe anaemia affected 12.1% of all women. Anaemia was more prevalent (p<0.001) among women who were aged 35–49 years (61.2%), were living in urban areas (73.2%), had higher education (63.0%), used bottled water (65.5%), were rich (65.1%), were underweight (66.9%), gave birth to ≥3 children (62.1%), were not currently pregnant (58.6%), were fecund (58.9%), or among those who menstruated in the last six weeks (59.9%).

**Table 3:** Characteristics of 15-49-year-old women participating in the 2016 Maldives Demographic and Health Survey.

General Characteristics	Total N (%)	Prevalence of Anaemia, N (%)			P*-*Value
		Moderate/severe	Mild	No Anaemia	
**Current Age**	6837				<0.001
15–24 Years	1869 (27.3)	203 (24.5)	263 (31.8)	361 (43.7)	
25–34 Years	2539 (37.1)	263 (10.4)	1220 (48.1)	1056 (41.6)	
35–49 Years	2429 (35.5)	361 (14.9)	1124 (46.3)	944 (38.9)	
**Place of Residence**	6837				<0.001
Urban	806 (11.8)	148 (18.4)	442 (54.8)	216 (26.8)	
Rural	6031 (88.2)	679 (11.3)	2731 (45.3)	2621 (43.5)	
**Education Level**	6837				<0.001
No Education	320 (4.7)	54 (16.9)	142 (44.4)	124 (38.8)	
Primary	1888 (27.6)	250 (13.2)	871 (46.1)	767 (40.6)	
Secondary	3598 (52.6)	375 (10.4)	1658 (46.1)	1565 (43.5)	
Higher	1031 (15.1)	148 (14.4)	502 (48.7)	381 (37.0)	
**Occupation**	6837				0.007
Agricultural	22 (0.3)	3 (13.6)	8 (36.4)	11 (50.0)	
Professional/Services	534 (7.8)	71 (13.3)	249 (46.6)	214 (40.1)	
Other	2445 (35.8)	330 (13.5)	1164 (47.6)	951 (38.9)	
Don’t Work	3836 (56.1)	423 (11.0)	1752 (45.7)	1661 (43.3)	
**Husband’s Education Level**	5021				0.067
No Education	913 (18.2)	65 (13.7)	223 (47.1)	185 (39.1)	
Primary	1605 (32.0)	211 (13.1)	749 (46.7)	645 (40.2)	
Secondary	2009 (40.0)	220 (11.0)	929 (46.2)	860 (42.8)	
Higher	494 (9.8)	77 (15.6)	232 (47.0)	185 (37.4)	
**Husband’s Occupation**	6837				0.308
Agricultural	925 (13.5)	111912.0)	416 (45.0)	398 (43.0)	
Non-Agricultural	1417 (20.7)	171 (12.1)	672 (47.4)	574 (40.5)	
Professional/Services	669 (9.8)	93 (13.9)	284 (42.5)	292 (43.6)	
Other/Don’t Work	3826 (56.0)	452 (11.8)	1801 (47.1)	1573 (41.1)	
**Sources of Drinking Water**	6837				<0.001
Piped Water	1002 (14.7)	119 (11.9)	468 (46.7)	415 (41.5)	
Rain Water	4632 (67.7)	532 (11.5)	2097 (45.3)	2003 (43.2)	
Bottled Water	1094 (16.0)	163 (14.9)	554 (50.6)	377 (34.5)	
Other	109 (1.6)	13 (11.95)	54 (49.5)	42 (38.5)	
**Toilet Facilities**	6837				0.551
Flush Toilet	6755 (98.8)	815 (12.1)	3132 (46.4)	2808 (41.6)	
Pit Latrine	12 (0.2)	2 (0.2)	4 (33.3)	6 (50.0)	
No Toilet	70 (1.0)	10 (14.3)	37 (52.9)	23 (32.9)	
**Household Wealth Status**	6837				<0.001
Poor	3829 (56.0)	439 (11.5)	1746 (45.6)	1644 (42.9)	
Medium	1863 (27.3)	206 (11.1)	864 (46.4)	793 (42.6)	
Rich	1145 (16.7)	182 (15.9)	563 (49.2)	400 (34.9)	
**BMI**	6837				<0.001
Underweight (<18.5 kg/m^2^)	644 (9.4)	97 (15.1)	333 (51.7)	214 (33.2)	
Normal (18.5–24.99 kg/m^2^)	2615 (38.2)	331 (12.7)	1254 (48.0)	1030 (39.4)	
Overweight/Obese (≥25.0 kg/m^2^)	3578 (52.4)	399 (11.2)	1586 (44.3)	1593 (44.5)	
**Total Number of Children Ever Birthed**	6837				<0.001
≥3 Children	2014 (29.5)	289 (14.3)	962 (47.8)	763 (37.9)	
1–2 Children	2817 (41.2)	313 (11.1)	1325 (47.0)	1179 (41.9)	
No Children	2006 (29.3)	225 (11.2)	886 (44.2)	895 (44.6)	
**Age at First Birth**	4831				0.002
15–24 Years	3831 (79.3)	477 (12.5)	1841 (48.1)	1513 (39.5)	
25–49 Years	1000 (20.7)	122 (12.7)	432 (44.8)	410 (42.5)	
**Current Contraception Use**	6837				0.001
Female Sterilization	251 (3.7)	46 (18.3)	131 (52.2)	74 (29.5)	
Using Other Method	760 (11.1)	91 (12.0)	356 (46.8)	313 (41.2)	
Not Using any Method	5826 (85.2)	690 (11.8)	2686 (46.1)	2450 (42.1)	
**Birth in the Last 3 Years**	6837				0.001
Yes	1503 (22.0)	140 (9.3)	717 (47.7)	646 (43.0)	
No	5334 (78.0)	687 (12.9)	2456 (46.0)	2191 (41.1)	
**Current Pregnancy Status**	6837				<0.001
Yes	255 (3.7)	54 (21.2)	91 (35.7)	110 (43.1)	
No	6582 (96.3)	773 (11.7)	3082 (46.8)	2727 (41.4)	
**Last Birth by Caesarean Section**	2356				0.194
Yes	964 (40.9)	83 (8.6)	464 (48.1)	417 (43.3)	
No	1392 (59.1)	151 (10.8)	662 (47.6)	579 (41.6)	
**Currently Breastfeeding**	6837				0.001
Yes	1110 (16.2)	99 (8.9)	514 (46.3)	497 (44.8)	
No	5727 (83.8)	728 (12.7)	2659 (46.4)	2340 (40.9)	
**Smoking Cigarettes**	6837				0.547
Yes	60 (0.9)	9 (15.0)	30 (50.0)	21 (35.0)	
No	6777 (99.1)	818 (12.1)	3143 (46.4)	2816 (41.6)	
**Fertility Status**	6837				<0.001
Fecund	4844 (70.0)	573 (11.8)	2280 (47.1)	1991 (41.1)	
Pregnant	255 (3.7)	54 (21.2)	91 (35.7)	110 (43.1)	
Postpartum Amenorrhoeic	250 (3.7)	21 (8.4)	107 (42.8)	122 (48.8)	
Infecund, Menopausal	1488 (21.8)	179 (12.0)	695 (46.7)	614 (41.3)	
**Ever had a Terminated Pregnancy**	6837				0.596
Yes	986 (14.4)	120 (12.2)	471 (47.8)	395 (40.1)	
No	5851 (85.6)	707 (12.1)	2702 (46.2)	2442 (41.7)	
**Menstruated in the Last 6 Weeks**	6837				<0.001
Yes	5477 (80.1)	673 (12.3)	2607 (47.6)	2197 (40.1)	
No	1360 (19.9)	154 (11.3)	566 (41.6)	640 (47.1)	

P-value obtained from Chi-square test.

The multiple multinomial logistic analyses in Table 4 showed that the following factors increased the odds of developing moderate/severe anaemia: residence in urban areas compared to residence in rural areas (aOR = 2.98, 95% CI: 1.93–4.68; p<0.001); underweight compared to normal weight (aOR = 1.96, 95% CI: 1.20–3.19; p = 0.004); having undergone female sterilization compared to not using any contraceptive methods (aOR = 1.71, 95% CI: 1.13–2.59; p = 0.011); current pregnancy compared to not being currently pregnant (aOR = 3.47, 95% CI: 2.11–5.69; p<0.001); and menstruation in the last six weeks compared to no menstruation in the last six weeks (aOR = 1.53, 95% CI: 1.14–1.91; p = 0.005). Overweight/obesity was associated with reduced odds of moderate/severe anaemia compared to normal weight (aOR = 0.64, 95% CI: 0.53–0.78; p<0.001). Factors associated with increased odds of mild anaemia (p<0.05) were residence in urban areas compared to residence in rural areas; underweight compared to normal weight; having undergone female sterilization compared to not using any contraceptive methods; or menstruation in the last six weeks compared to no menstruation in the last six weeks. Women from rich households, overweight/obese women, and women who gave birth to their first child at the age of 25–49 years had reduced odds of mild anaemia (p<0.05) compared to women from households of medium wealth, women with normal weight, and women who gave birth to their first child before 25 years of age, respectively (Table 4).

**Table 4:** Factors associated with anaemia among women of reproductive age in Maldives (n = 6837).

General Characteristics	Moderate/severe Anaemia				Mild Anaemia			
	cOR [95% CI]	P-Value	aOR [95% CI]	P-Value	cOR [95% CI]	P-Value	aOR [95% CI]	P-Value
**Current Age**								
15–24 Years (Ref.)	1.00		1.00		1.00		1.00	
25–34 Years	0.63 [0.53–0.78]	<0.001	1.66 [0.98–2.70]	0.051	0.83 [0.74–0.95]	0.005	1.34 [0.98–1.82]	0.068
35–49 Years	0.65 [0.55–0.78]	<0.001	1.15 [0.73–1.74]	0.536	0.97 [0.87–1.09]	0.621	1.29 [1.00–1.67]	0.050
**Place of Residence**								
Rural (Ref.)	1.00		1.00		1.00		1.00	
Urban	2.65 [2.11–3.31]	<0.001	2.98 [1.93–4.68]	<0.001	1.96 [1.66–2.22]	<0.001	2.46 [1.79–3.38]	<0.001
**Education Level**								
Secondary (Ref.)	1.00		1.00		1.00		1.00	
No Education	1.82 [1.30–2.55]	0.001	1.13 [0.75–1.71]	0.553	0.87 [0.67–1.15]	0.318	0.87 [0.65–1.17]	0.369
Primary	1.36 [1.13–1.63]	0.001	0.93 [0.71–1.21]	0.586	0.86 [0.74–1.02]	0.077	0.88 [0.74–1.06]	0.171
Higher	1.62 [1.30–2.02]	<0.001	1.13 [0.83–1.55]	0.170	0.80 [0.69–0.94]	0.004	1.15 [0.94–1.41]	0.178
**Sources of Drinking Water**								
Other (Ref.)	1.00		1.00		1.00		1.00	
Piped Water	0.93 [0.48–1.78]	0.819	1.01 [0.39–2.60]	0.987	0.88 [0.58–1.34]	0.545	0.71 [0.40–1.26]	0.247
Rain Water	0.86 [0.46–1.61]	0.634	1.15 [0.46–2.90]	0.768	0.81 [0.55–1.22]	0.814	0.74 [0.43–1.28]	0.281
Bottled Water	1.40 [0.74–2.67]	0.313	1.25 [0.48–3.25]	0.645	1.14 [0.75–1.75]	0.537	0.88 [0.49–1.56]	0.655
**Household Wealth Status**								
Medium (Ref.)	1.00		1.00		1.00		1.00	
Poor	1.75 [1.39–2.21]	<0.001	0.94 [0.75–1.17]	0.558	1.29 [1.10–1.62]	0.002	0.97 [0.84–1.12]	0.699
Rich	1.03 [0.85–1.24]	0.771	0.92 [0.63–1.35]	0.676	0.98 [0.87–1.10]	0.670	0.77 [0.59–0.99]	0.042
**BMI**								
Normal (18.5–24.99 kg/m^2^) (Ref.)	1.00		1.00		1.00		1.00	
Underweight (<18.5 kg/m^2^)	1.41 [1.08–1.85]	0.013	1.96 [1.20–3.19]	0.004	1.23 [1.06–1.55]	0.012	1.90 [1.34–2.71]	<0.001
Overweight/Obese (≥25.0 kg/m^2^)	0.78 [0.66–0.92]	0.003	0.64 [0.53–0.78]	<0.001	0.82 [0.73–0.91]	<0.001	0.76 [0.66–0.86]	<0.001
**Total Number of Children Ever Birthed**								
No Children (Ref.)	1.00		1.00		1.00		1.00	
≥3 Children	1.51 [1.23–1.84]	<0.001	1.26 [0.98–1.61]	0.069	1.27 [1.12–1.46]	<0.001	1.05 [0.49–2.26]	0.900
1–2 Children	1.10 [0.88–1.28]	0.578	0.80 [0.23–2.85]	0.724	1.14 [1.01–1.28]	0.041	0.94 [0.46–1.95]	0.862
**Age at First Birth**								
15–24 Years (Ref.)	1.00		1.00		1.00		1.00	
25–49 Years	1.26 [1.10–1.51]	0.010	0.90 [0.70–1.17]	0.427	1.24 [1.10–1.39]	<0.001	0.78 [0.66–0.93]	0.004
**Current Contraception Use**								
Not Using any Method (Ref.)	1.00		1.00		1.00		1.00	
Female Sterilization	2.21 [1.51–3.22]	<0.001	1.71 [1.13–2.59]	0.011	1.62 [1.21–2.16]	0.001	1.49 [1.09–2.03]	0.012
Using Other Method	1.03 [0.81–1.32]	0.802	0.96 [0.73–1.26]	0.758	1.04 [0.88–1.22]	0.655	0.95 [0.79–1.13]	0.533
**Birth in the Last 3 Years**								
No (Ref.)	1.00		1.00		1.00		1.00	
Yes	0.69 [0.57–0.85]	<0.001	0.83 [0.60–1.16]	0.316	0.99 [0.88–1.12]	0.873	1.08 [0.88–0.14]	0.471
**Current Pregnancy Status**								
No (Ref.)	1.00		1.00		1.00		1.00	
Yes	1.73 [1.24–2.42]	0.001	3.47 [2.11–5.69]	<0.001	0.73 [0.55–0.97]	0.300	1.15 [0.78–1.68]	0.485
**Currently Breastfeeding**								
No (Ref.)	1.00		1.00		1.00		1.00	
Yes	0.64 [0.51–0.81]	<0.001	0.82 [0.57–1.18]	0.284	0.91 [0.80–1.04]	0.172	0.85 [0.68–1.10]	0.142
**Fertility Status**								
Infecund, Menopausal (Ref.)	1.00		1.00		1.00		1.00	
Fecund	0.99 [0.82–1.20]	0.895	1.00 [0.81–1.27]	0.970	1.01 [0.89–1.15]	0.854	1.02 [0.87–1.21]	0.783
Pregnant	1.68 [1.17–2.43]	0.005	3.47 [2.11–5.69]	<0.001	0.73 [0.55–0.98]	0.039	1.15 [0.78–1.68]	0.485
Postpartum Amenorrhoeic	0.59 [0.37–0.97]	0.036	1.15 [0.66–2.24]	0.635	0.78 [0.60–1.03]	0.076	0.97 [0.69–1.36]	0.848
**Menstruated in the Last 6 Weeks**								
No (Ref.)	1.00		1.00		1.00		1.00	
Yes	1.27 [1.05–1.55]	0.016	1.53 [1.14–1.91]	0.005	1.34 [1.18–1.52]	<0.001	1.22 [1.01–1.47]	0.035

Ref: Reference; CI: Confidence Interval.

P-value obtained from multiple multinomial logistic regression models.

Of the 6419 women of reproductive age in Nepal, 39.1% were aged 15–24 years, and 64.4% resided in urban areas (Table 5). The overall prevalence of anaemia in Nepal was 40.6%, while moderate/severe anaemia was present among 6.9% of all women and mild anaemia was present in 33.7% of all women. Anaemia was more prevalent (p<0.001) among women who were aged 15–24 years (43.5%), were Muslim (51.1%), had sources of drinking water other than tube well or tap water (49.2%), had no toilet (50.9%), came from households of medium wealth (47.5%), were underweight (47.2%), underwent female sterilization (53.5%), gave birth in the last three years (45.5%), were currently pregnant (43.3%), did not smoke (41.4%), or had postpartum amenorrhoea (45.7%).

**Table 5:** Characteristics of 15-49-year-old women participating in the 2016 Nepal Demographic and Health Survey.

General Characteristics	Total N (%)	Prevalence of Anaemia, N (%)			P-Value
		Moderate/severe	Mild	No Anaemia	
**Current Age**	6419				<0.001
15–24 Years	2512 (39.1)	185 (7.4)	906 (36.1)	1421 (56.6)	
25–34 Years	1910 (29.8)	134 (7.0)	648 (33.9)	1128 (59.1)	
35–49 Years	1997 (31.1)	132 (6.6)	603 (30.2)	1262 (63.2)	
**Place of Residence**	6419				0.741
Urban	4134 (64.4)	298 (7.2)	1385 (33.5)	2451 (59.3)	
Rural	2285 (35.6)	153 (6.6)	772 (33.8)	1360 (59.5)	
**Religion**	6419				<0.001
Hindu	5590 (87.1)	401 (7.2)	1888 (33.8)	3301 (59.1)	
Buddhist	306 (4.8)	16 (5.2	79 (25.8)	211 (69.0)	
Islam	282 (4.4)	22 (7.8)	122 (43.3)	138 (48.9)	
Kirat	76 (1.2)	4 (5.3)	17 (22.4)	55 (72.4)	
Christian	165 (2.6)	8 (4.8)	51 (30.9)	106 (64.2)	
**Education Level**	6419				0.052
No Education	2173 (33.9)	160 (7.4)	736 (33.9)	1277 (58.8)	
Primary	1020 (15.9)	73 (7.2)	314 (30.8)	633 (62.1)	
Secondary	2333 (36.3)	164 (7.0)	828 (35.5)	1341 (57.5)	
Higher	893 (13.9)	54 (6.0)	279 (31.2)	560 (62.7)	
**Occupation**	6419				0.002
Agricultural	3200 (49.9)	200 (6.3)	1059 (33.1)	1941 (60.7)	
Professional/Services	787 (12.3)	55 (7.0)	237 (30.1)	495 (62.9)	
Other	406 (6.3)	24 (5.9)	142 (35.0)	240 (59.1)	
Don’t Work	2026 (31.6)	172 (8.5)	719 (35.5)	1135 (56.0)	
**Husband’s Education Level**	4936				0.009
No Education	761 (15.4)	64 (8.4)	286 (37.6)	411 (54.0)	
Primary	1134 (23.0)	84 (7.4)	342 (30.2)	708 (62.4)	
Secondary	2165 (43.9)	139 (6.4)	211 (32.8)	1315 (60.7)	
Higher	876 (17.7)	66 (7.5)	290 (33.1)	520 (59.4)	
**Husband’s Occupation**	6419				0.003
Agricultural	1089 (17.0)	71 (6.5)	368 (33.8)	650 (59.7)	
Business	1652 (25.7)	129 (7.8)	585 (35.4)	938 (56.8)	
Professional/Services	1600 (24.9)	110 (6.9)	473 (29.6)	1017 (63.6)	
Other/Don’t Work	2078 (32.4)	141 (6.8)	731 (35.2)	1206 (58.0)	
**Sources of Drinking Water**	6419				<0.001
Tap Water	2121 (33.0)	114 (5.4)	663 (26.5)	1444 (68.1)	
Tube Well	1190 (18.5)	59 (5.0)	342 (28.7)	789 (66.3)	
Other	3108 (48.4)	278 (8.9)	1252 (40.3)	1578 (50.8)	
**Toilet Facilities**	6419				<0.001
Flush Toilet	4777 (74.4)	322 (6.7)	1492 (31.2)	2963 (62.0)	
Pit Latrine	518 (8.1)	36 (6.9)	201 (38.8)	281 (54.2)	
Other	425 (6.6)	31 (7.3)	170 (40.0)	224 (52.7)	
No Toilet	699 (10.9)	62 (8.9)	294 (42.1)	343 (49.1)	
**Household Wealth Status**	6419				<0.001
Poor	2666 (41.5)	159 (6.0)	819 (30.7)	1688 (63.3)	
Medium	1320 (20.6)	104 (7.9)	523 (39.6)	693 (52.5)	
Rich	2433 (37.9)	188 (7.7)	815 (33.5)	1430 (58.8)	
**BMI**	6419				<0.001
Underweight (<18.5 kg/m^2^)	1060 (16.5)	87 (8.2)	415 (39.0)	562 (52.8)	
Normal (18.5–24.99 kg/m^2^)	4095 (63.8)	304 (7.3)	1450 (34.7)	2426 (58.0)	
Overweight/Obese (≥25.0 kg/m^2^)	1264 (19.7)	46 (5.5)	199 (23.9)	589 (70.6)	
**Total Number of Children Ever Birthed**	6419				0.200
≥3 Children	2203 (34.3)	153 (6.9)	730 (33.15)	1320 (59.9)	
1–2 Children	2385 (37.2)	156 (6.5)	785 (32.9)	1444 (60.5)	
No Children	1831 (28.5)	142 (7.8)	642 (35.1)	1047 (28.5)	
**Age at First Birth**	4588				0.246
15–24 Years	4266 (93)	283 (6.8)	1388 (33.2)	2511 (60.0)	
25–49 Years	322 (7.0)	18 (5.7)	94 (29.8)	203 (64.4)	
**Current Contraception Use**	6419				<0.001
Female Sterilization	688 (10.7)	78 (11.3)	290 (42.2)	320 (46.5)	
Using Other Method	1901 (29.6)	95 (5.0)	533 (28.0)	1273 (67.0)	
Not Using any Method	3830 (59.7)	278 (7.3)	1334 (34.8)	2218 (57.9)	
**Birth in the Last 3 Years**	6419				<0.001
Yes	1386 (21.6)	100 (7.2)	531 (38.3)	755 (54.5)	
No	5033 (78.4)	351 (7.0)	1626 (32.3)	3056 (60.7)	
**Current Pregnancy Status**	6419				<0.001
Yes	289 (4.5)	48 (16.6)	77 (26.6)	164 (56.7)	
No	6130 (95.5)	403 (6.6)	2080 (33.9)	3647 (59.5)	
**Last Birth by Caesarean Section**	2020				0.620
Yes	181 (9.0)	16 (8.8)	65 (35.9)	100 (55.2)	
No	1839 (91.0)	127 (6.9)	665 (36.2)	1047 (56.9)	
**Currently Breastfeeding**	6419				0.003
Yes	1479 (23.0)	103 (7.0)	551 (37.3)	825 (55.8)	
No	4940 (77.0)	348 (7.0)	1606 (32.5)	2986 (60.0)	
**Smoking Cigarettes**	6419				<0.001
Yes	424 (6.6)	24 (5.7)	101 (23.8)	299 (70.5)	
No	5995 (93.4)	427 (7.1)	2056 (34.3)	3512 (58.6)	
**Fertility Status**	6419				<0.001
Fecund	5087 (79.2)	329 (6.5)	1751 (34.4)	3007 (59.1)	
Pregnant	289 (4.5)	48 (16.6)	77 (26.0)	164 (56.7)	
Postpartum Amenorrhoeic	324 (5.0)	26 (8.0)	122 (37.7)	176 (54.3)	
Infecund, Menopausal	719 (11.2)	48 (6.7)	207 (28.7)	464 (64.5)	
**Ever had a Terminated Pregnancy**	6419				0.543
Yes	1333 (20.8)	95 (7.1)	431 (32.3)	807 (60.5)	
No	5086 (79.2)	356 (7.0)	1726 (33.9)	3004 (59.1)	
**Menstruated in the Last 6 Weeks**	6419				0.058
Yes	5117 (79.7)	344 (6.7)	1747 (34.1)	3026 (59.1)	
No	1302 (20.3)	1079 (8.2)	410 (31.5)	785 (60.3)	

P-value obtained from Chi-square test.

Factors associated with increased odds of moderate/severe anaemia included having undergone female sterilization compared to not using any contraceptive methods (aOR = 2.04, 95% CI: 1.45–2.86; p<0.001); or current pregnancy compared to not being currently pregnant (aOR = 2.72, 95% CI: 1.68–4.43; p<0.001) (Table 6). Factors associated with decreased odds of moderate/severe anaemia were agricultural occupation compared to not being currently employed (aOR = 0.77, 95% CI: 0.61–0.98; p = 0.037); living in a household with tap water (aOR = 0.57, 95% CI: 0.44–0.73; p<0.001) or tube well (aOR = 0.55, 95% CI: 0.40–0.76; p<0.001) as the source of drinking water compared to other sources of drinking water; and overweight/obesity compared to normal weight (aOR = 0.60, 95% CI: 0.44–0.80; p = 0.001) (Table 6). Factors associated with increased odds of mild anaemia (p<0.05) were having undergone female sterilization compared to not using any contraceptive methods; having given birth in the last three years compared to not having given birth in the last three years; and being fecund compared to being infecund/menopausal. Factors associated with decreased odds of mild anaemia (p<0.05) were tap water or tube well as the source of drinking water compared to other sources of drinking water; living in a poor household compared to living in a household of medium wealth; and using methods of contraception other than sterilization compared to not using any methods of contraception. Overweight/obesity was associated with lower odds of mild anaemia compared to normal weight. In addition, women with primary or higher education were less likely to be affected by mild anaemia compared to women with secondary education. Cigarette smoking was associated with lower odds of mild anaemia compared to not smoking (aOR = 0.64, 95% CI: 0.50–0.83; p = 0.001) (Table 6).

**Table 6:** Factors associated with anaemia among women of reproductive age in Nepal (n = 6419).

General Characteristics	Moderate/severe Anaemia				Mild Anaemia			
	cOR [95% CI]	P-Value	aOR [95% CI]	P-Value	cOR [95% CI]	P-Value	aOR [95% CI]	P-Value
**Current Age**								
15–24 Years (Ref.)	1.00		1.00		1.00		1.00	
25–34 Years	0.80 [0.64–1.02]	0.069	0.97 [0.68–1.38]	0.861	0.75 [0.66–0.85]	<0.001	1.01 [0.83–1.22]	0.903
35–49 Years	0.91 [0.72–1.16]	0.446	1.03 [0.78–1.36]	0.850	0.90 [0.79–1.02]	0.109	1.07 [0.92–1.25]	0.392
**Religion**								
Christian (Ref.)	1.00		1.00		1.00		1.00	
Hindu	1.61 [0.80–3.33]	0.199	1.43 [0.68–2.98]	0.346	1.19 [0.85–1.67]	0.317	1.12 [0.80–1.61]	0.530
Buddhist	1.01 [0.42–2.42]	0.992	1.15 [0.47–2.81]	0.758	0.78 [0.51–1.19]	0.245	0.91 [0.59–1.41]	0.681
Islam	2.11 [0.91–4.93]	0.084	1.18 [0.49–2.82]	0.712	1.84 [1.22–2.78]	0.004	1.25 [0.83–1.97]	0.310
Kirat	0.96 [0.28–3.34]	0.953	1.05 [0.30–3.70]	0.940	0.64 [0.34–1.22]	0.174	0.68 [0.35–1.34]	0.248
**Education Level**								
Secondary (Ref.)	1.00		1.00		1.00		1.00	
No Education	1.03 [0.81–1.29]	0.837	0.98 [0.72–1.33]	0.871	1.16 [0.98–1.37]	0.093	0.86 [0.73–1.02]	0.091
Primary	0.94 [0.71–1.26]	0.693	0.94 [0.69–1.29]	0.719	1.00 [0.82–1.21]	0.966	0.79 [0.66–0.94]	0.008
Higher	0.79 [0.57–1.09]	0.149	0.76 [0.54–1.07]	0.113	1.24 [1.05–1.50]	0.012	0.83 [0.69–0.99]	0.036
**Occupation**								
Don’t Work (Ref.)	1.00		1.00		1.00		1.00	
Agricultural	0.68 [0.55-.844]	<0.001	0.77 [0.61–0.98]	0.037	0.86 [0.76–0.97]	0.014	0.93 [0.82–1.07]	0.323
Professional/Services	0.73 [0.53–1.01]	0.058	1.07 [0.76–1.51]	0.742	0.76 [0.63–0.91]	0.002	1.04 [0.85–1.26]	0.724
Other	0.66 [0.42–1.03]	0.070	0.75 [0.47–1.17]	0.197	0.93 [0.74–1.17]	0.556	1.05 [0.82–1.32]	0.703
**Husband’s Occupation**								
Other/Don’t Work (Ref.)	1.00		1.00		1.00		1.00	
Agricultural	0.93 [0.69–1.26]	0.658	1.04 [0.72–1.49]	0.860	0.93 [0.80–1.09]	0.396	1.15 [0.95–1.40]	0.164
Business	1.18 [0.91–1.52]	0.209	1.11 [0.82–1.50]	0.487	1.03 [0.90–1.18]	0.686	1.06 [0.89–1.25]	0.491
Professional/Services	0.93 [0.71–1.20]	0.562	0.92 [0.68–1.26]	0.610	0.77 [0.67–0.89]	<0.001	0.86 [0.73–1.02]	0.089
**Sources of Drinking Water**								
Other (Ref.)	1.00		1.00		1.00		1.00	
Tap Water	0.45 [0.36–0.56]	<0.001	0.57 [0.44–0.73]	<0.001	0.49 [0.44–0.56]	<0.001	0.61 [0.53–0.70]	<0.001
Tube Well	0.42 [0.32–0.57]	<0.001	0.55 [0.40–0.76]	<0.001	0.55 [0.47–0.63]	<0.001	0.67 [0.57–0.79]	<0.001
**Toilet Facilities**								
No Toilet (Ref.)	1.00		1.00		1.00		1.00	
Flush Toilet	0.60 [0.45–0.81]	0.001	0.98 [0.70–1.35]	0.864	0.59 [0.50–0.70]	<0.001	0.85 [0.71–1.03]	0.095
Pit Latrine	0.71 [0.46–1.10]	0.125	1.05 [0.65–1.64]	0.835	0.84 [0.66–1.06]	0.138	1.12 [0.87–1.44]	0.373
Other	0.77 [0.48–1.22]	0.258	0.83 [0.51–1.36]	0.461	0.89 [0.69–1.14]	0.346	0.93 [0.71–1.22]	0.610
**Household Wealth Status**								
Medium (Ref.)	1.00		1.00		1.00		1.00	
Poor	0.63 [0.48–0.82]	0.001	0.83 [0.63–1.10]	0.203	0.64 [0.56–0.74]	<0.001	0.78 [0.67–0.91]	0.001
Rich	0.88 [0.68–1.13]	0.311	1.08 [0.82–1.44]	0.574	0.76 [0.66–0.87]	<0.001	0.92 [0.79–1.08]	0.296
**BMI**								
Normal (18.5–24.99 kg/m^2^) (Ref.)	1.00		1.00		1.00		1.00	
Underweight (<18.5 kg/m^2^)	1.25 [0.96–1.61]	0.093	1.18 [0.91–1.54]	0.222	1.24 [1.07–1.43]	0.003	1.11 [0.96–1.29]	0.165
Overweight/Obese (≥25.0 kg/m^2^)	0.61 [0.46–0.80]	<0.001	0.60 [0.44–0.80]	0.001	0.60 [0.52–0.69]	<0.001	0.65 [0.55–0.76]	<0.001
**Current Contraception Use**								
Not Using any Method (Ref.)	1.00		1.00		1.00		1.00	
Female Sterilization	1.95 [1.47–2.57]	<0.001	2.04 [1.45–2.86]	<0.001	1.51 [1.23–1.80]	<0.001	1.45 [1.18–1.78]	<0.001
Using Other Method	0.60 [0.47–0.76]	<0.001	0.78 [0.58–1.03]	0.083	0.70 [0.62–0.79]	<0.001	0.77 [0.66–0.89]	<0.001
**Birth in the Last 3 Years**								
No (Ref.)	1.00		1.00		1.00		1.00	
Yes	1.15 [0.91–1.45]	0.237	1.12 [0.70–1.79]	0.640	1.32 [1.17–1.50]	<0.001	1.40 [1.08–1.83]	0.013
**Current Pregnancy Status**								
No (Ref.)	1.00		1.00		1.00		1.00	
Yes	2.65 [0.89–3.71]	<0.001	2.72 [1.68–4.43]	<0.001	0.82 [0.64–1.09]	0.167	0.89 [0.63–1.25]	0.495
**Currently Breastfeeding**								
No (Ref.)	1.00		1.00		1.00		1.00	
Yes	1.07 [0.85–1.35]	0.563	0.97 [0.61–1.54]	0.893	1.24 [1.10–1.41]	0.001	0.93 [0.72–1.21]	0.604
**Smoking Cigarettes**								
No (Ref.)	1.00		1.00		1.00		1.00	
Yes	0.66 [0.43–1.01]	0.057	0.74 [0.47–1.17]	0.199	0.58 [0.46–0.73]	<0.001	0.64 [0.50–0.83]	0.001
**Fertility Status**								
Infecund, Menopausal (Ref.)	1.00		1.00		1.00		1.00	
Fecund	1.06 [0.77–1.45]	0.730	1.07 [0.76–1.51]	0.713	1.31 [1.10–1.55]	0.003	1.28 [1.05–1.54]	0.011
Pregnant	2.83 [1.83–4.38]	<0.001	2.72 [1.68–4.43]	<0.001	1.05 [0.77–1.44]	0.752	0.89 [0.63–1.25]	0.495
Postpartum Amenorrhoeic	1.43 [0.86–2.37]	0.169	1.27 [0.71–2.28]	0.419	1.55 [1.17–2.06]	0.002	1.10 [0.81–1.55]	0.505

Ref: Reference; CI: Confidence Interval.

P-value obtained from multiple multinomial logistic regression models.

## Discussion

This study reported the prevalence of and factors associated with anaemia among women of reproductive age in three South-Asian countries using recent nationally-representative data. The overall prevalence of anaemia was 41.8% in Bangladesh, 58.5% in Maldives, and 40.6% in Nepal. The high prevalence of anaemia among women in Bangladesh, Maldives and Nepal are in line with previous studies [15, 27, 28]. However, the prevalence of anaemia found in this study is higher than the global prevalence of anaemia of 32.8% in 2016 [29].

Our findings align with previous studies examining factors associated with anaemia at a global level and in the South-Asian region. Factors associated with greater odds of anaemia in our study were low BMI, sterilization, current pregnancy, low education level, having given birth in the last three years, and menstruation in the last six weeks whereas having access to safe water or hygienic toilets were found to protect against anaemia.

Women of reproductive age are more vulnerable to anaemia due to blood loss associated with menstruation and childbirth and due to haemodilution during pregnancy. In addition, the low socioeconomic status in this region compared with other parts of the world may contribute to poor dietary intakes and thus high prevalence of micronutrient deficiencies in addition to high occurrence of infectious and parasitic diseases, and haemoglobinopathies which all can contribute to the development of anaemia. National nutritional programmes such as iron and folic acid supplementation, homestead food production programs and other programmes implemented by governments and stakeholders might have contributed to reducing anaemia prevalence in Bangladesh and Nepal in the last two decades [30] although further efforts to address this problem are clearly needed.

Women with low BMI (<18.5 kg/m^2^) had higher odds of anaemia which is consistent with previous studies in South-Asian countries [4, 10, 15, 27, 31]. This may be due to undernourished women lacking necessary micronutrients which may lead to anaemia. Provision of micronutrient programmes for disadvantaged women of reproductive age should therefore be considered. In this study, overweight and obesity were found to be associated with lower odds of anaemia compared to normal weight. However, dissimilar results have been documented in previous research where overweight and obesity were found to be a risk factor for anaemia among women of reproductive age [32, 33]. A possible explanation may be that overweight/obese women in our study might have had greater nutrient intakes compared to underweight/normal weight women that could have resulted in greater Hb concentrations. For example, a study carried out in Chinese women noted that overweight/obese women had higher iron intakes than normal weight women [32] which may be due to higher energy consumption thus higher micronutrient intake.

Using contraceptive methods other than sterilization showed a strong association with decreased odds of anaemia. A study in Nepal highlighted that the use of Depo-Provera injections had a significant positive impact on Hb concentrations in women [34]. Reduction of menstrual bleeding linked with the use of hormonal contraceptives could be the possible underlying mechanism. Low level of education was associated with increased odds of anaemia. The plausible explanation could be that a low level of education prevents women from obtaining employment or leads to poorly paid employment which may result in low income. This limits household earnings and reduces the purchasing ability of good quality and quantity of foods and routine medical check-up, and may also lead to infections and parasitic diseases, which are often associated with poverty. Our findings are consistent with previous studies in low- and middle-income countries, showing increased risk of anaemia among women living in poor families [10, 22]. This is in contrast to other studies carried out in Nepal and Pakistan that reported negative associations of wealth with anaemia [22, 35]. Several studies have also shown that higher education increases the odds of becoming mildly anaemic [27, 36, 37] while our findings disagree with previous studies because we noted that higher education decreased the risk of being mildly anaemic.

Multiple periods of breastfeeding of successive children, repeated pregnancies, and menstruation in the last six weeks have also been found to be associated with anaemia [15, 28]. In addition, current pregnancy was found as an important risk factor for anaemia among women in all three countries in our study. Anaemia during pregnancy is a global burden and 56% of pregnant women have been reported to be at risk of anaemia in low-income countries [38]. Pregnant women are more prone to anaemia [23] due to physiological changes such as increases in blood volume, and increased demand for iron and other micronutrients due to fetal demand [39].

Having access to safe sources of drinking water (i.e., tap water and tube wells) was found as a marginally protective factor for reducing anaemia among women in Nepal and Bangladesh. Using unhygienic toilets was associated with an increased chance of anaemia because unhygienic toilets may contribute to parasitic diseases which can lead to chronic blood loss as a result of gastrointestinal parasite infestation [15].

The strength of this study is the inclusion of the most current nationally-representative survey data. The limitations include a cross-sectional design, therefore no causal relationship between anaemia and predictor variables can be established. Secondly, anaemia can be caused by malaria, parasitic infections, infectious diseases, genetic haemoglobin disorders, or inadequate dietary intake, however this information was not available in the data sources. Thus, there is a need for longitudinal studies, and inclusion of a comprehensive list of potential factors associated with anaemia in future research.

## Conclusions

Our findings show that anaemia remains at an unacceptable level in Bangladesh, Maldives, and Nepal despite improvements in education, sanitation and other socioeconomic conditions. There is an urgent need for effective policies and programmes to reduce the burden of anaemia in these three South-Asian countries. All future programmes developed to prevent and control anaemia in Bangladesh, Maldives and Nepal should target the at-risk groups identified in this study.
